# Epigenetic-Transcriptional Regulation of Fatty Acid Metabolism and Its Alterations in Leukaemia

**DOI:** 10.3389/fgene.2018.00405

**Published:** 2018-09-24

**Authors:** Michael Maher, Jeannine Diesch, Raquel Casquero, Marcus Buschbeck

**Affiliations:** ^1^Josep Carreras Leukaemia Research Institute (IJC), Campus ICO-Germans Trias i Pujol-Universitat Autònoma de Barcelona, Barcelona, Spain; ^2^Program for Predictive and Personalized Medicine of Cancer, Germans Trias i Pujol Research Institute (PMPPC-IGTP), Barcelona, Spain

**Keywords:** cancer, AML, fatty acid metabolism, fatty acid oxidation, epigenetics, CPT1, transcription

## Abstract

In recent years fatty acid metabolism has gained greater attention in haematologic cancers such as acute myeloid leukaemia. The oxidation of fatty acids provides fuel in the form of ATP and NADH, while fatty acid synthesis provides building blocks for cellular structures. Here, we will discuss how leukaemic cells differ from healthy cells in their increased reliance on fatty acid metabolism. In order to understand how these changes are achieved, we describe the main pathways regulating fatty acid metabolism at the transcriptional level and highlight the limited knowledge about related epigenetic mechanisms. We explore these mechanisms in the context of leukaemia and consider the relevance of the bone marrow microenvironment in disease management. Finally, we discuss efforts to interfere with fatty acid metabolism as a therapeutic strategy along with the use of metabolic parameters as biomarkers.

## Introduction

Acute myeloid leukaemia (AML) is a group of disorders affecting the myeloid lineage of blood cells in the bone marrow. Blood cells are formed in the bone marrow where they originate from haematopoietic stem cells (HSC). In AML, HSCs undergo genetic mutations that result in ineffective haematopoiesis and dysfunctional blood cells due to impaired differentiation (Shih et al., 2012). These leukaemia stem cells show spontaneous apoptosis *in vitro* but increased proliferation *in vivo* (Lane et al., 2009), indicating that intrinsic factors of the cells and extrinsic factors in the bone marrow environment may contribute to their survival. AML cells can be characterised by aberrant genetic and epigenetic changes that distinguish them from healthy cells (Metzeler et al., 2016; Wouters and Delwel, 2016; Fisher et al., 2017). It has been well documented that cancer cells, including AML, can also be distinguished metabolically from normal cells (Kohli and Passegué, 2014; Berger et al., 2017). Otto Warburg proposed that cancer cells exhibit increased glycolysis in the presence of oxygen (Warburg effect), thereby providing the cells with a more readily accessible source of ATP (Warburg, 1956). Initially, this idea led researchers to think of cancer in terms of metabolic dysfunction due to mitochondrial injury. Instead, what is becoming evident is that metabolic plasticity may be a cellular adaption to increased energy demands of proliferating cells in a harsh tumour microenvironment in which there may be limited nutrient and oxygen supply. These unfavourable conditions require cancer cells to modulate their metabolism to one that promotes survival and proliferation, which in turn may lead to drug resistance (Ma et al., 2018).

Dysregulation of fatty acid (FA) metabolism has been implicated in a variety of diseases and a prominent role in cancer is emerging. FA synthesis is required for anabolic reactions such as membrane biosynthesis and generation of signalling molecules. From the oxidation of FAs, ATP yield is more than twice that of glucose or amino acids, making FAs an important fuel. This review endeavours to highlight the changes in lipid metabolism that distinguish malignant AML cells from normal, healthy cells. Firstly, to give some background, we provide a summary of anabolic and catabolic FA metabolism and an overview of key transcriptional regulators. We also present and discuss relevant epigenetic regulators and the reciprocal effects of FA metabolism on epigenetic mechanisms.

## FA metabolism

### FA synthesis and the storage of high-energy fuel

**Figure 1:**
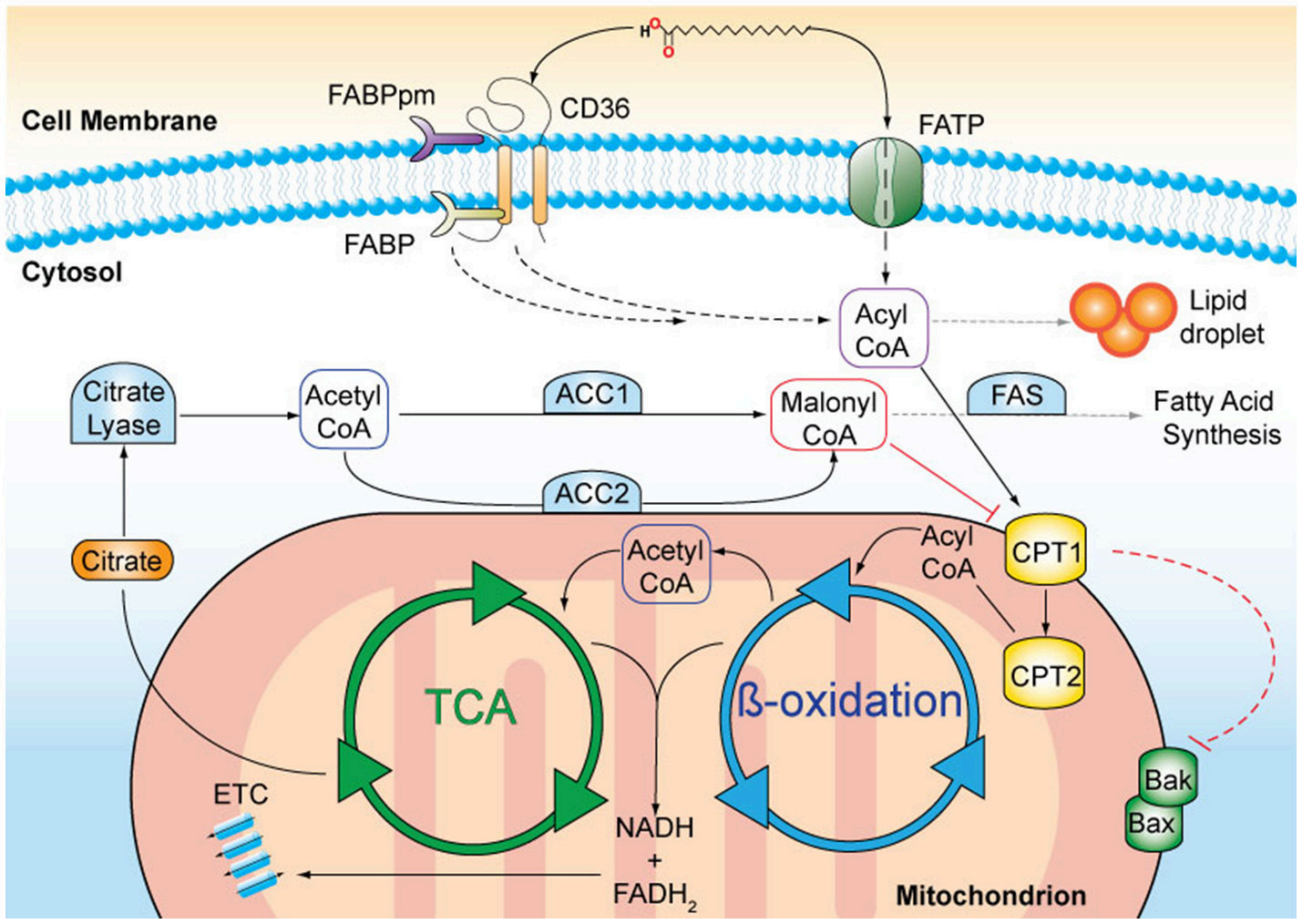
A schematic representation of fatty acid (FA) metabolism. Long chain FAs, such as palmitic acid, are actively transported across the cell membrane by membrane-bound transporters, such as CD36 and FA transport protein (FATP). FA binding proteins (FABPpm, membrane-associated; FATP, cytosolic) facilitate the transfer. In the cytosol, FAs can either be stored in lipid droplets or undergo enzymatic conversion to FA-acyl-CoA that can enter the mitochondria via the carnitine palmitoyltransferases 1 and 2 (CPT1, CPT2) transporters, located on the outer and inner mitochondrial membranes, respectively. The activation of CPT1 is a survival signal and inhibits the oligomerisation of the pro-apoptotic Bcl-2 family proteins, Bak, and Bax. Inside mitochondria, FA-acyl-CoA molecules are broken down in a series of enzymatic reactions known as ß-oxidation. FADH_2_ and NADH are released and are used as co-factors in the electron transport chain (ETC) to produce ATP. Acetyl-CoA is released and enters the tricarboxylic acid cycle (TCA), where it is oxidised for citrate production. Citrate is transported to the cytosol where it is converted to acetyl-CoA. Acetyl-CoA carboxylase 1 (ACC1) –mediated conversion of acetyl-coA to malonyl-CoA is the rate-limiting step in fatty acid synthesis. Malonyl-CoA in particular when produced by ACC2, inhibits CPT1 and thus limits ß-oxidation.

Lipids originate from dietary sources or are generated by *de novo* FA biosynthesis occurring mainly in the liver and adipose tissue (reviewed in Salati and Goodridge, 1996). Acetyl-CoA is the precursor of FA synthesis and is produced in the mitochondria from FA oxidation (Figure 1). Acetyl-CoA is converted within the tricarboxylic acid (TCA) cycle to citrate and subsequently transported into the cytoplasm by the citrate transporter. In the cytoplasm, citrate is cleaved by citrate lyase regenerating acetyl-CoA that can then be used for FA synthesis. The first and rate-limiting step of FA synthesis is the ATP-dependent carboxylation of acetyl-CoA to malonyl-CoA catalyzed by acetyl-CoA-carboxylase 1 (ACC1). The remaining steps are catalyzed by the FA synthase (FAS) complex, which leads to a series of reactions until the 16-carbon FA palmitic acid, is synthesised. Further elongation and desaturation takes place at the endoplasmatic reticulum membrane (Salati and Goodridge, 1996).

ACC exists in two isoforms, ACC1 and ACC2 (Abu-Elheiga et al., 1997), and is activated by citrate and inhibited by palmitoyl-CoA and malonyl-CoA by allosteric regulation (Trumble et al., 1995). Further, both ACC isoforms are phosphorylated by AMP-activated protein kinase (AMPK), an important cellular sensor of low energy states, which leads to their inhibition (Munday et al., 1988; Winder et al., 1997). Conversely, prolyl hydroxylase 3 (PHD3) activates ACC2 via proline hydroxylation (German et al., 2016). In addition to the short-term and transient regulation through post-translational modifications, long-term mechanisms include changes in expression of genes encoding key FA synthesis enzymes and occur in response to dietary factors. For instance, consuming a carbohydrate-rich diet increases ACC1 and FAS expression, which then promotes FA formation (Kim, 1997). Conversely, fasting decreases FA synthesis by inhibiting ACC1 and FAS expression (Pape et al., 1988).

### ß-oxidation

FAs are degraded by ß-oxidation in the mitochondria providing energy in the form of ATP and acetyl-CoA for protein acetylation and anabolic reactions. Several membrane-associated proteins including CD36, membrane-associated FA-binding proteins (FABP) and a number of FA transport proteins facilitate FA uptake into the cell (Stremmel et al., 2001). In particular, CD36 plays an important role in the regulation of FA uptake due to its ability to translocate between intracellular endosomes and the plasma membrane. This intracellular translocation is dependent on FA availability, the energy status of the cell (Luiken et al., 2003) as well as CD36 transcriptional activation (Bastie et al., 2005). Once in the cell, FAs undergo conversion into long-chain acyl-CoA catalysed by fatty acyl-CoA synthase. Acyl-CoA is transported into the mitochondria by the carnitine palmitoyltransferases, CPT1 and CPT2, that are located at the outer and inner mitochondrial membranes, respectively (McGarry et al., 1977). Acyl-CoA is subsequently converted into acetyl-CoA through ß-oxidation, which then enters the TCA cycle (Kunau et al., 1995). ß-oxidation is inversely coupled to FA synthesis and regulated by ACC2-derived malonyl-CoA, which inhibits mitochondrial FA uptake by CPT1. Conversely, malonyl-CoA decarboxylase decreases the inhibition of CPT1 by decarboxylating malonyl-CoA to acetyl-CoA, leading to an elevated rate of FA oxidation (McGarry et al., 1977; Ruderman and Dean, 1998). Importantly, ß-oxidation enzymes are susceptible to negative feedback inhibition in which the intermediates they produce inhibit their activity (Kunau et al., 1995).

### Transcriptional regulation of FA metabolism

**Figure 2:**
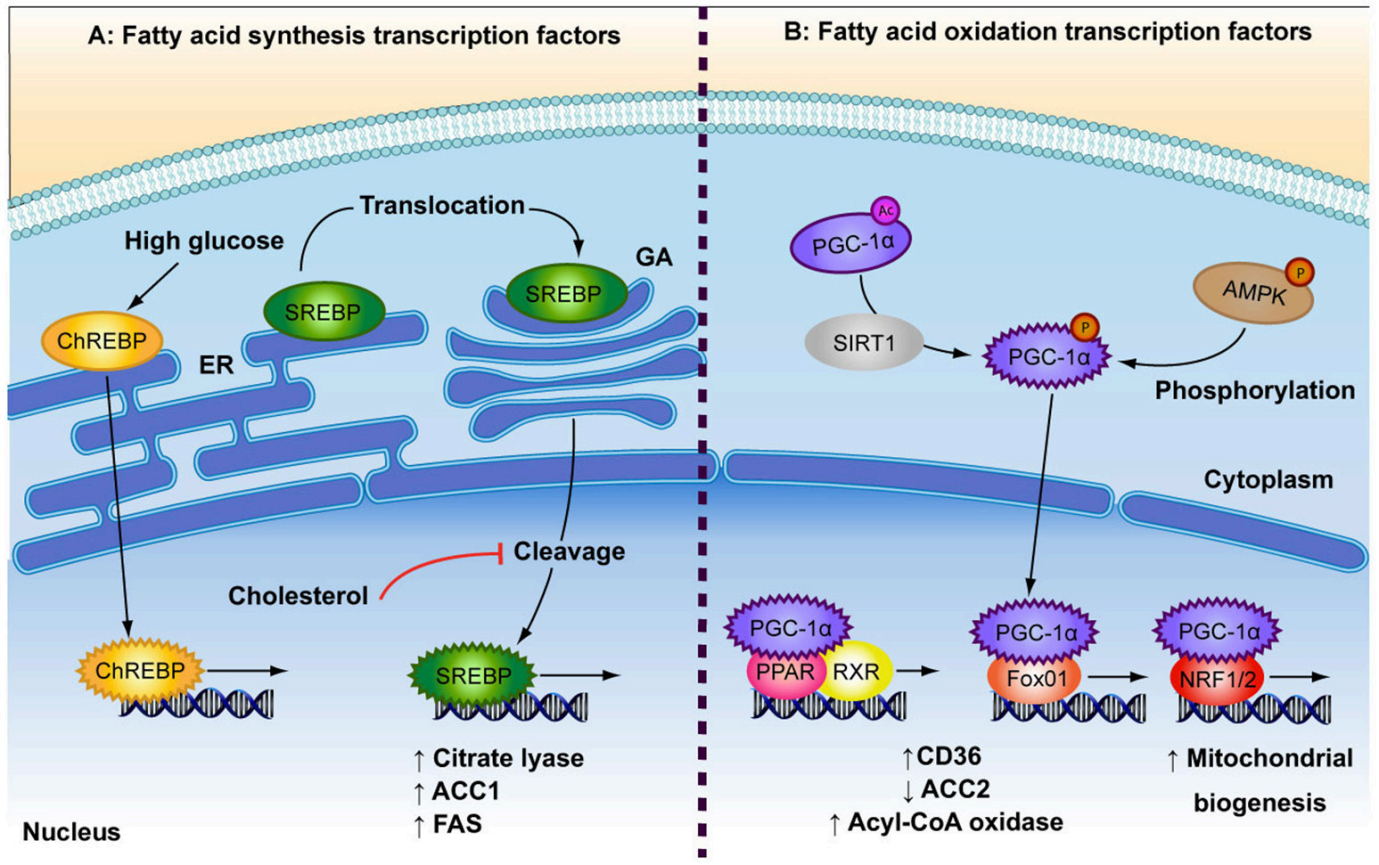
Depicted are the main transcription factors involved in fatty acid (FA) synthesis and FA oxidation. **(A)** In response to high glucose concentrations, carbohydrate responsive element-binding protein (ChREBP) is transported to the nucleus. Sterol regulatory element-binding protein (SREBP) is bound to the endoplasmic reticulum (ER) where it is translocated to the golgi apparatus (GA). SREBP is cleaved to produce its active transcription factor form, a process that is inhibited by high levels of cholesterol. Both ChREBP and SREBP are involved in FA synthesis by increasing expression of citrate lyase, acetyl-CoA carboxylase (ACC1) and fatty acid synthase (FAS). **(B)** The low energy sensors, AMPK and SIRT1, activate peroxisome proliferator-activated receptor-gamma coactivator 1 alpha (PGC-1α), which after translocation to the nucleus, interacts with several transcription factors: Peroxisome proliferator-activated receptor (PPAR) and retinoid X receptor (RXR), which heterodimerise upon ligand binding; forkhead box protein O1 (Fox01); and nuclear respiratory factor 1 (NRF1/2). These events result in up-regulation of FA oxidation by increasing expression of FA transporters and rate-limiting enzymes (CD36, ACC2, acyl-CoA oxidase) and by increasing overall mitochondrial biogenesis.

Both anabolic and catabolic processes of FA metabolism are under the control of transcription factors (TFs) (Figure 2). Peroxisome proliferator-activated receptors (PPAR) are key TFs involved in increased FA oxidation (reviewed in Poulsen et al., 2012). PPARs act as heterodimers with retinoid X receptor (RXR) and are activated by binding DNA and FA ligands (Forman et al., 1997; Aagaard et al., 2011; Poulsen et al., 2012). PPARα and PPARß/δ are involved in increased FA uptake and activation of mitochondrial ß-oxidation in various cell types, while PPARγ is mainly expressed in adipose tissue and is a potent inducer of adipogenesis (reviewed in Poulsen et al., 2012). FA metabolism genes that are induced by PPARs include CD36, FATP, acyl-CoA synthetase, malonyl-CoA decarboxylase and CPT1 (reviewed in Desvergne and Wahli, 1999; Mandard et al., 2004). Likewise, peroxisome proliferator-activated receptor-gamma co-activator alpha (PGC-1α) is involved in increased overall FA oxidation as well as mitochondrial biogenesis. AMPK and sirtuin 1 (SIRT1) activate PGC-1α through phosphorylation and deacetylation, respectively, in response to low energy sources (Rodgers et al., 2005; Jäger et al., 2007). PGC-1α is a potent activator and a target of several other metabolism-related TFs involved in the up-regulation of oxidative metabolism, including PPARα and PPAR∂ (Vega et al., 2000), forkhead box protein O1 (FOXO1; Puigserver et al., 2003), nuclear respiratory factors (NRF1 and NRF2; Wu et al., 1999), as well as the lipogenic regulator, carbohydrate-responsive element-binding protein (ChREBP; Chambers et al., 2013). FOXO1 enhances FA oxidation by increasing expression of acyl-CoA oxidase and PPARδ, repressing ACC2 and by promoting FA uptake through translocation of CD36 to the plasma membrane (Bastie et al., 2005). NRF1 and NRF2 are principal promoters of mitochondrial biogenesis and thus increase the β-oxidation capacity of the cell (Scarpulla, 1997). NRF1 further regulates several FA oxidation regulators such as PPARα, Lipin1, and PGC-1ß (Hirotsu et al., 2012).

Anabolic regulators of FA metabolism play a role in countering the effects of higher oxidation in times of plentiful nutrient supply by increasing FA synthesis and storage. A build up of FAs or cholesterol can be toxic to cells and so feedback loops are in place to control intracellular levels. Sterol regulatory element-binding proteins (SREBP) are required for the control of *de novo* FA and cholesterol synthesis (Bengoechea-Alonso and Ericsson, 2007; Espenshade and Hughes, 2007). SREBPs are bound to the endoplasmic reticulum from where they translocate to the nucleus in response to depleted intracellular FA or cholesterol levels (Sakai et al., 1996). The SREBP-1c isoform has been shown to upregulate the expression of several genes involved in FA synthesis, including citrate lyase, ACC1, and FAS (reviewed in Foufelle and Ferré, 2002). ChREBP is a glucose-responsive TF. Glucose enhances ChREBP nuclear translocation and DNA binding by decreasing phosphorylation (Kawaguchi et al., 2001), while FAs inhibit ChREBP activity (Dentin et al., 2005). Glucose has been shown to induce ChREBP gene expression in the liver (Dentin et al., 2004), which in turn induces lipogenic genes such as ACC1 and FAS (Ishii et al., 2004). Taken together, both FA synthesis and ß-oxidation are regulated on the transcriptional level by a range of opposingly acting TFs and dietary cues.

### Interplay between epigenetic regulation and fatty acid metabolism

While TF-mediated regulation in metabolism is generally transient, epigenetic factors may confer prolonged alterations, which can be transmitted to the next generation. Chromatin modifications comprise the molecular basis of epigenetic mechanisms, of which DNA methylation is related with gene silencing (reviewed in Wolffe and Matzke, 1999), and histone acetylation is associated with gene transcription (Marmorstein and Zhou, 2014). Isocitrate dehydrogenase 1 (IDH1) mutations are implicated in AML and other myeloid malignancies. IDH1 inhibition results in reduced α-ketoglutarate (αKG) production, leading to increased histone methylation, which has been shown to increase tumour cell differentiation and increase cells' therapy response (Calvert et al., 2017). Diets rich in fat have been shown to affect chromatin accessibility of regulatory gene regions in rodents (Leung et al., 2014). Several studies in rodent offspring have shown that higher maternal dietary fat intake caused persistent DNA hypermethylation and down-regulation of the *Fads2* gene, which encodes FA desaturase in FA synthesis (Niculescu et al., 2011, 2013; Kelsall et al., 2012). Similar diet-induced epigenetic changes found in adult rodents could be reversed by decreasing fat intake (Hoile et al., 2013). Maternal high-fat diet has also been reported to induce hypermethylation of the PGC-1α promoter in skeletal muscle cells. Interestingly, the resulting decreased expression can be counteracted by maternal exercise, further highlighting the plasticity of FA metabolism (Laker et al., 2014).

Acetyl-CoA is generated from glucose via glycolysis and is substrate for histone acetylation (Takahashi et al., 2006). Indeed, high levels of glucose have been shown to increase histone acetylation (Wellen and Thompson, 2012), while a converse reduction in acetyl-CoA synthesis results in rapid histone deacetylation (Takahashi et al., 2006). In this way, acetyl-CoA is an important link between energy metabolism and chromatin regulation (Rathmell and Newgard, 2009; Wellen and Thompson, 2012). FAs also affect acetyl-CoA levels and thus histone acetylation. On the one hand, *de novo* FA synthesis uses acetyl-CoA as substrate, and therefore competes with histone acetylation for the same acetyl-CoA pool. Lowering the rate of FA synthesis, by reducing ACC1 expression, increases global histone acetylation and gene expression (Galdieri and Vancura, 2012). On the other hand, stimulating FA oxidation, and thereby increasing acetyl-CoA levels, leads to increased histone acetylation (McDonnell et al., 2016). In addition epigenetic factors may also act on non-chromatin substrates to regulate FA metabolism. This has been observed in the histone deacetylase 3 (HDAC3) and SIRT1 inhibition of PPARγ (Qiang et al., 2012; Jiang et al., 2014).

Interestingly, metabolic enzymes can also more directly act to bring about changes in chromatin structure and gene transcription. AMPK has been shown to phosphorylate H2B histones to activate transcription of AMPK-responsive genes, such as CPT1c, during metabolic stress (Bungard et al., 2010). Similarly, AMPK phosphorylation of the methytransferase enzyme, EZH2, represses polycomb repressive complex 2 (PRC2) -mediated methylation, thereby up-regulating tumour suppressor genes (Wan et al., 2018). Further, it has been reported that almost all glycolytic enzymes are RNA-binding proteins, thereby linking metabolism and gene transcription (Beckmann et al., 2015).

Overall there is complementary interplay between epigenetic regulation and FA metabolism that is mediated by dietary FAs directly altering methylation states and by the provision of acetyl-CoA for acetylation.

## The role of FA metabolism in leukaemia

### The role of epigenetic regulation in altered FA metabolism

It is now well accepted that epigenetic changes contribute to haematological cancers (Pastore and Levine, 2016). Altered DNA methylation patterns are a hallmark of AML, partly due to dysregulation of DNA methyltransferase (DNMT)-encoding genes (reviewed in Wu et al., 1999). During recent years links between epigenetic regulation and an altered FA metabolism have been emerging in AML. For instance, FABP4 has a dual role in increasing FA uptake and signalling to the epigenetic regulators, which together create a favourable environment for AML proliferation (Yan et al., 2017). Mechanistically, FABP4 up-regulation increases IL-6 expression and STAT3 phosphorylation leading to DNMT1 overexpression and silencing of the cell cycle inhibitor, p15 (Yan et al., 2017). Conversely, forced DNMT1 expression caused increased FABP4 expression in AML, pointing towards a possible metabolic-epigenetic feedback loop (Yan et al., 2018). The epigenetic silencing of the *ACC2* gene is a key step that drives the reliance of AML cells on FA oxidation. Repression of the *ACC2* gene by SIRT1-dependent histone deacetylation allows for simultaneous ß-oxidation and FA synthesis to take place (Corbet and Feron, 2017). Simultaneous up-regulation of lipolysis and dysregulation of lipogenesis has been speculated to be a potential hallmark of cancer cell metabolism (Carracedo et al., 2013). Indeed, the ACC2 activator, PHD3, has been shown to be down-regulated in around 80% of AML patients, resulting in higher ß-oxidation (German et al., 2016).

Healthy haematopoietic and leukaemia stem cells have been traditionally identified by immunophenotyping cell markers (Bennett et al., 1976). However, metabolic heterogeneity among these cell populations is becoming increasingly evident. CD36-positive leukaemia stem cells were shown to have elevated FA uptake and ß-oxidation (Ye et al., 2016). In addition to increased FA uptake, higher rates of anaerobic glycolysis contribute to increased ß-oxidation in AML cells by promoting a decreased electrochemical gradient on the mitochondrial membrane and uncoupling of the electron transport chain (Samudio et al., 2010). While we are just at the beginning of understanding the significance of metabolic changes in leukaemia, increased reliance on FAs as fuel is becoming apparent.

### The bone marrow is the nutrient-providing HSC niche

The bone marrow microenvironment provides nutrients and growth signals to both healthy HSCs and disease clones. The bone marrow is composed of an array of different cell types including adipocytes and mesenchymal stem cells and is the pertinent site of interest in leukaemia (Medyouf, 2017). Adipocytes account for approximately 70% of the tissue mass of the bone marrow (Hardaway et al., 2014). AML blasts undergo spontaneous apoptosis *in vitro*, but proliferate *in vivo* in the bone marrow (Lane et al., 2009), indicating that the bone marrow environment contributes to extrinsic growth-promoting factors. Indeed, bone marrow adipocytes protect acute monocytic leukaemia cells by disrupting apoptosis. Adipocytes supply leukaemia cells with FA ligands that induce PPARγ-controlled FA oxidation genes, thereby promoting cell survival (Tabe et al., 2017). Further evidence of metabolic cross-talk involves secreted FABP4 proteins that act as carrier proteins for FA transport between adipocytes and AML blasts (Shafat et al., 2017). Adipocytes also produce adipokines such as leptin and adiponectin, which modulate FA metabolism of nearby cells (VanSaun, 2013). Interestingly, AML cells also exhibit higher rates of ß-oxidation when co-cultured with mesenchymal stem cells (Samudio et al., 2008). A mouse study showing that osteoblast cells induce leukaemogenesis in HSCs via Fox01 (Kode et al., 2016). Overall, AML cells manage to take advantage of the robust growth-promoting environment of the bone marrow.

### Interference with therapy

Allogeneic haematopoietic cell transplantation (alloHCT) remains the only curative option for AML (Bejanyan et al., 2015). However, due to the invasive nature of alloHCT and compounding risk factors of comorbidities, chemotherapies remain the preferred treatment options for elderly AML patients (Ustun et al., 2013). Recent studies have indicated that metabolic changes may confer drug resistance. High oxidative phosphorylation has been associated with cytarabine (ara-C)–resistance in leukaemia cells (Farge et al., 2017). Although Ara-C killed both resting and proliferating cancer cells, the remaining resistant cells were characterised by increased FA oxidation and up-regulated CD36. In another study, CD36-positive leukaemia cells were shown to be relatively more drug-resistant to AraC *in vivo* and *in vitro* compared with CD36-negative cells (Ye et al., 2016). Moreover, high expression of CD36 and CPT1a recorded in different cohorts of AML patients was associated with poor prognosis and shorter overall survival (Perea et al., 2005; Shi et al., 2016). Although predicting AML patient response to drugs based on cellular metabolic profiles remains elusive, these observations point towards a key role of FA metabolism, particularly increased β-oxidation. This might explain why obesity is a leading risk factor for most cancers (Lichtman, 2010). In the context of leukaemia, excess adipose tissue increases the risk of disease onset (Naveiras et al., 2009) and is associated with poorer outcome due to chemotherapy resistance (Behan et al., 2009; Ye et al., 2016). Coupled with these findings is the increased proportion of adipose tissue in the bone marrow as people age, which incidentally correlates with increased rates of disease incidence (Stenderup et al., 2003). Taken together, the accumulation of bone marrow adipose tissue and incidence of obesity represent probable risk factors for acquiring AML and subsequent therapy resistance.

Based on these findings, efforts have been made to target FA metabolism as a therapeutic strategy. For instance, the FA uptake protein CD36 has been evaluated as a potential target. Sulfo-N-succinimidyl oleate (SSO) is a FA analogue that inhibits CD36 function (Kuda et al., 2013) and has been shown to perturb cell growth *in vitro* (Coort et al., 2002) and reduce CPT1 activity (Campbell et al., 2004). However, its toxicity *in vivo* deems SSO unsuitable for therapeutic use. As an alternative strategy, inhibitory CD36-specific antibodies increase sensitivity of chronic myelogenous leukaemia cells to the first-line drug imatinib (Landberg et al., 2018). Etomoxir irreversibly inhibiting CPT1 and thus ß-oxidation (Abdel-aleem et al., 1994) and has been successfully used for the treatment of cardiac conditions (Bristow, 2000). In AML, etomoxir sensitises cells to apoptosis-inducing treatments (Samudio et al., 2010). Another CPT1 inhibitor, ST1326, was shown to inhibit proliferation, survival and chemoresistance in leukaemia cell lines and primary cells by driving cells to apoptosis and causing toxic accumulation of cytosolic palmitate (Ricciardi et al., 2015). Collectively, these studies indicate that inhibition or reversal of increased FA oxidation has been shown to be a suitable therapeutic intervention, in particular when combined with other cytotoxic drugs.

## Outlook and conclusion

FA metabolism is up-regulated in many cancer types, such as colorectal (Zhou et al., 2013), ovarian (Wang et al., 2005), and glioblastoma (Beckner et al., 2009). Metabolic adaptations of leukaemia cells to the microenvironment contribute to proliferation and disease progression (Samudio et al., 2010). Cancer cells develop resistance in part by increasing FA oxidation and thus, not surprisingly, obesity is emerging as a major risk factor. This provides rational for supportive therapeutic measures through nutritional intervention.

At present, it is not clear to which extent metabolic adaptations of cancer cells are either stable or transient. Future investigations will need to explore how epigenetic mechanisms regulate and sustain metabolic states in healthy cells and also how cancers cells adapt to their microenvironment. Promising initial studies that have investigated the dependence of cancer cells on FA oxidation warrant follow-up in pre-clinical models, in particular as part of combinatorial therapies.

## Author contributions

MM and JD wrote the main body of the text. MM and RC designed and illustrated the figures. MM, JD, and MB participated in redrafting of the manuscript and contributed feedback to the final manuscript. All authors have approved the manuscript for submission.

### Conflict of interest statement

The authors declare that the research was conducted in the absence of any commercial or financial relationships that could be construed as a potential conflict of interest.
